# Iron-rich food consumption and associated factors among children aged 6–23 months in sub-Saharan Africa: A multilevel analysis of Demographic and Health Surveys

**DOI:** 10.1371/journal.pone.0253221

**Published:** 2021-06-17

**Authors:** Yonas Akalu, Yigizie Yeshaw, Getayeneh Antehunegn Tesema, Getu Debalkie Demissie, Meseret Derbew Molla, Abebe Muche, Mengistie Diress, Sofonyas Abebaw Tiruneh

**Affiliations:** 1 Department of Physiology, School of Medicine, College of Medicine and Health Sciences, University of Gondar, Gondar, Ethiopia; 2 Department of Epidemiology and Biostatistics, Institute of Public Health, College of Medicine and Health Sciences, University of Gondar, Gondar, Ethiopia; 3 Department of Health Education and Behavioral Sciences, Institute of Public Health, College of Medicine and Health Sciences, University of Gondar, Gondar, Ethiopia; 4 Department of Biochemistry, School of Medicine, College of Medicine and Health Sciences, University of Gondar, Gondar, Ethiopia; 5 Department of Human Anatomy, School of Medicine, College of Medicine and Health Sciences, University of Gondar, Gondar, Ethiopia; 6 Department of Public Health, College of Health Sciences, Debre Tabor University, Debre Tabor, Ethiopia; Universidade de Sao Paulo Faculdade de Saude Publica, BRAZIL

## Abstract

**Introduction:**

Anemia remains a major public health problem for children in sub-Saharan Africa (SSA). Iron-rich foods consumption has a determinant role on the anemia status. Hence, this study aimed to determine the prevalence of good consumption of iron-rich foods and its associated factors among children aged 6–23 months in SSA.

**Materials and methods:**

The recent Demographic and Health Survey data sets of thirty-five SSA countries were used. Data were analyzed using STATA/MP version 16.0 and all statistical analyses were done after weighting the data. A generalized linear mixed model using Poisson regression with robust error variance was used to determine factors associated with good consumption of iron-rich food. Association of variables was declared at a p-value of ≤0.05 and adjusted prevalence ratio (aPR) ratio with its 95% confidence interval (CI) was calculated for each variable.

**Results:**

The total weighted samples of 77,001 children aged 6–23 months were included. The prevalence of consumption of iron rich foods was 42.1% (95% CI: 41.78–42.48). Children with age of 12–17 (adjusted prevalence ratio (aPR) = 1.96, 95% CI: 1.89–2.04) and 18–23 months (aPR = 2.05, 95% CI: 1.97–2.14), who took drugs for intestinal parasites (aPR = 1.30, 95% CI: 1.26–1.34), with postnatal check within 2 months (aPR = 1.09, 95% CI: 1.06–1.13), and children from women with ANC visit of 1–3 (aPR = 1.31, 95% CI: 1.24–1.37) and ≥4 (aPR = 1.41, 95% CI: 1.34–1.48) had higher prevalence of good consumption of iron rich foods. Moreover, the prevalence of consumptions of iron rich foods was higher among children from; family with rich (aPR = 1.36, 95%CI: 1.30–1.42) and middle (aPR = 1.14 95% CI: 1.09–1.19) wealth index, and mother with media exposure (aPR = 1.26, 95%CI: 1.22–1.31).

**Conclusion:**

The prevalence of good consumption of iron-rich foods among children aged 6–23 months in SSA countries is low. Child factors, family factors, and community-level factors were significantly associated with consumption of iron rich foods. Strategies to increase the consumption of iron-rich foods during this critical stage of growth and development should be designed in SSA.

## Introduction

One in two under-five children suffer from hidden hunger (micronutrient deficiency) [1], and iron deficiency (ID) is the world’s commonest micronutrient deficiency affecting more than 2 billion people in the world, with the highest-burden in African children [2, 3]. This most widespread nutritional deficiency, ID, is the primary cause of iron deficiency anemia (IDA) (hemoglobin levels of < 11g/dl) [4, 5]. Almost half, 47% of under-five children are anemic, which is mainly attributed to ID [6].

The low oxygen-carrying capacity of blood in anemia leads to lack of oxygen supply to the fast-growing children’s brain and negatively affects mental, motor, and cognitive development, which in turn lead to social withdrawal, attention deficit, and impaired school performance of children [7–9]. Iron deficiency in the absence of anemia is even more frequent and has a similar negative impact on mental development which may be irreversible, especially in children less than 2 years, despite adequate therapy [10, 11]. In severe cases, iron deficiency anemia in children is associated with increased mortality [12] and heart failure [13].

According to the 2011 world health organization(WHO) report, anemia affects 60.2% of African children aged 6–59 months, with the highest prevalence in under-five children of sub-Saharan countries, ranging from 74 to 86%, and is primarily due to ID [6]. Iron deficiency in SSA countries is highly prevalent and ranges from 21.7% to 41.9% [14].

The most vulnerable groups to iron deficiency are under-two children who are in high iron demand due to the rapid growth and brain development. As a result, WHO recommends daily iron supplementation for this age group, 6–23 months, in areas where the prevalence of anemia is 40% or greater [15]. Moreover, numerous countries design preventive strategies and conduct interventions like iron supplementation and deworming. However, anemia remains a major public health problem in children in the world, especially in SSA [12].

The possible causes of ID during early childhood are increased iron needs due to rapid growth, inadequate iron intake due to exclusively breastfed without iron supplementation, inadequate dietary iron intake, low availability of dietary sources of iron secondary to low socioeconomic status, and dietary restrictions [16]. Thus, to prevent ID in children older than 6 months, the American Academy of Pediatrics and WHO recommend the introduction and consumption of two servings per day of iron-rich complementary foods, including iron-fortified cereals and pureed meats [17–19]. However, less than half (17–65%) of children consumed iron-rich foods regularly [20]. Iron-rich foods were not commonly consumed among infants in urban areas of China [21]. More than half of infants did not consume the recommended iron-rich food [22] and the consumption of meat, the main iron source, in Africa is the lowest in the world [23]. In French-speaking African countries and Ethiopia, consumption of iron-rich foods ranges from 17–65% [20, 24]. Some of the factors that affect children’s consumption of iron-rich foods include lack of awareness of mothers on iron-rich foods, child age, maternal education, religion, household wealth status, and feeding culture [23–25].

Though iron-rich foods consumption is the main determinant of anemia and a strategy targeted for adequate intake of iron could be achievable and improved [26], there has not been a continent-wide analysis that identified iron-rich foods consumption status and its determinants in children aged 6–23 months in sub-Saharan Africa. Therefore, this study aimed to determine the prevalence of good consumption of iron-rich foods and its associated factors among children aged 6–23 months in sub-Saharan Africa.

The information from this study will help for effective nutritional interventions and assist government and non-governmental organizations, health practitioners, and policymakers to design intervention strategies to improve consumption of iron-rich foods for the most vulnerable group in the most vulnerable sub-regions within SSA.

## Materials and methods

### Data source

We used the appended most recent nationally representative demographic and health survey (DHS) datasets of 35 sub-Saharan Africa countries. The DHS, collected every five years, is a nationally representative survey that provides population and health indicators at the regional and national levels. DHS surveys are designed to collect data on marriage, fertility, family planning, reproductive health, child health, nutrition, HIV/AIDS, and mortality. Pre-tested and standard DHS questionnaires were used for data collection of the DHS surveys. The questionnaire was conceptualized to the different countries context and the data were collected by trained data collectors [27]. The datasets were obtained from the measure DHS Program at https://www.dhsprogram.com/data/dataset_admin/login_main.cfm.

Those countries with no data on the outcome variable (Sierra Leone and Sudan) were excluded from the analysis. The total weighted samples of 77,001 children aged 6–23 months were included in the study.

### Variables of the study

#### Dependent variable

The dependent variable of this study was children’s (aged 6–23 months) consumption of iron-rich foods, dichotomized as good and poor. According to the DHS, consumption of at least one iron-rich food item among the four food items: meat (beef, pork, lamb, chicken, etc.), egg, organ meat(liver, heart, or other organs), and fish or shellfish at any time in the last 24 hours before the interview was considered as good consumption. On the other hand, no history of iron-rich food consumption in the last 24 hours period preceding the interview was declared as poor consumption [27].

#### Independent variables

**Fig 1:**
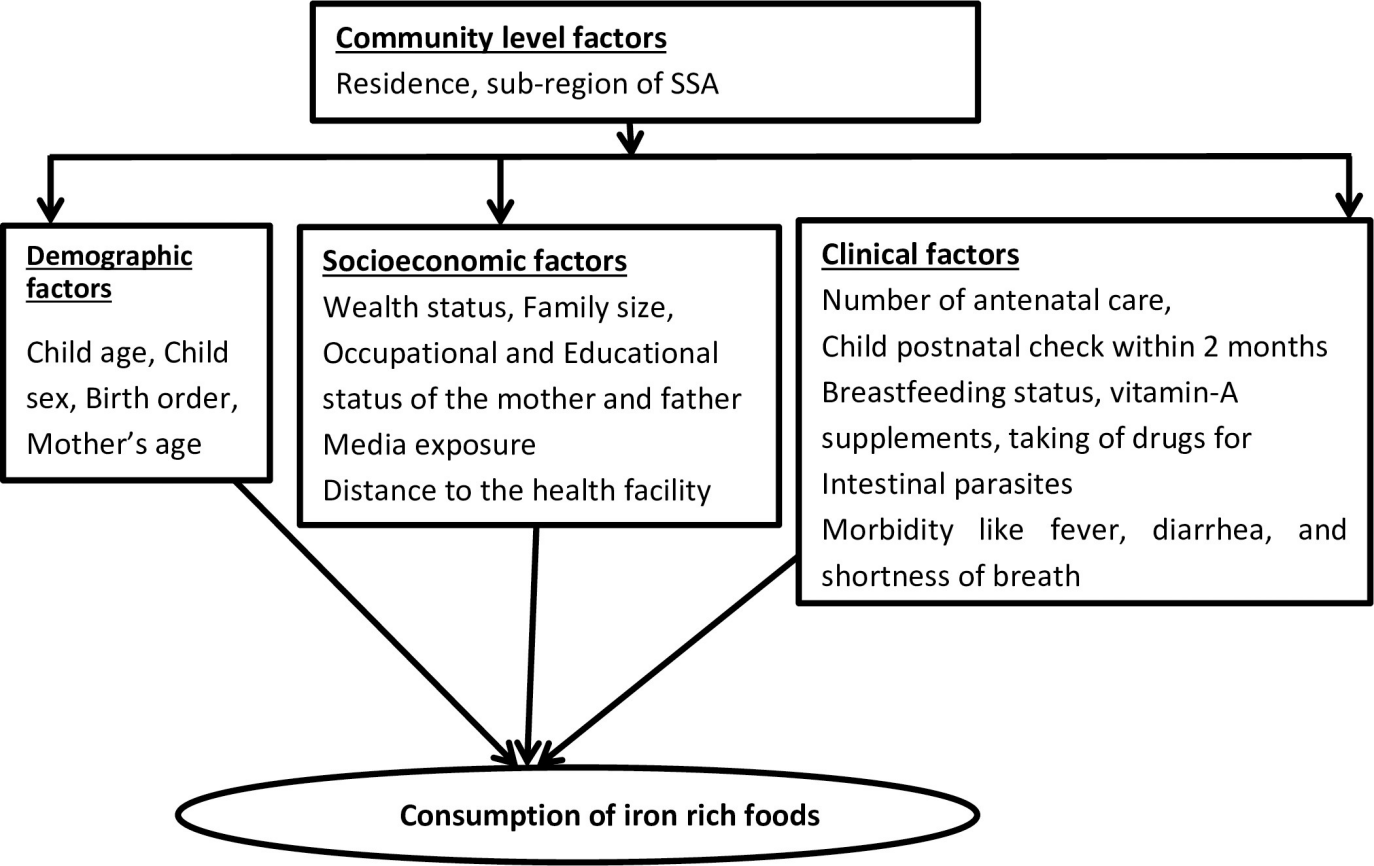
Conceptual framework for factors associated with consumption of iron rich foods in SSA.

This study included both individual and community-level variables. The individual-level variables were age (6–11, 12–17, and 18–23 months), sex (male or female), breastfeeding status of the child (yes or no), receiving vitamin-A supplements (yes or no), birth order (1, 2–4, and ≥5, mother’s age (<20, 20–34, 35–49 years), any morbidity like fever, diarrhea, and short breath(yes or no), taking of drugs for intestinal parasites (yes or no), educational status of the mother and father (no education, primary, secondary, and higher), occupational status of mother and father (working or not working), number of antenatal care (ANC) (not at all, 1–3, and 4–16), child postnatal check within 2 months (yes or no), family size (≤5 or >5), wealth status (poor, middle, and rich), distance to the health facility (big problem or not a big problem), and media exposure (media exposure was created from the three variables: watching television, listening radio, and reading a newspaper, and labeled as yes if a woman has exposure to either of the three media sources or no if a woman has exposure to none of them). Residence (urban or rural) and region of SSA (East Africa, West Africa, Central Africa, and southern Africa) were the community-level variables (Fig 1).

### Data analysis procedure

We used STATA version 16.0/MP software for analysis. Before doing any analysis, the data were weighted to ensure the representativeness of the DHS sample and get reliable estimates and standard error [27].

To account for the hierarchical nature of DHS data, since children are within household and household are within the cluster resulting in the correlation of observations which violate the assumption of independence, measures of community variation: Intra-class Correlation Coefficient (ICC); Median Odds Ratio (MOR) and Proportional Change in Variance (PCV) were estimated. We fitted four models: the null-model (without independent variables), model I (only individual-level variables), model II (only community-level variables), and model III (both the individual and community level variables). The smallest deviance value was observed in Model III and hence was the best-fitted model.

Since the outcome variable, good consumption of iron rich foods, is a common problem (42. 1%), a generalized linear mixed model using Poisson regression with robust error variance was employed to identify the associated factors of iron-rich foods consumption among children 6–23 months in SSA. We fitted the bi-variable and multivariable multilevel robust Poisson regression to select variables. So, we choose variables with p-value less than 0.25 in the bi-variable analysis for the multivariable analysis and we considered those clinically important variables. During the bi-variable analysis all the clinically important predictors which were available in the DHS data had p-value less than 0.25. So we have not missed important variables which were reported and assumed as significant predictors of iron rich food consumption. Therefore, we have used stepwise approach than backward elimination and forward variable selection methods considering the clinical significance. Moreover, to choose the variables we have used LLR and pseudo-R2-value to check whether the included variable can cause model improvement. In the multivariable multilevel robust Poisson regression analysis, we used the adjusted prevalence ratio (aPR) with 95% CI and p-value of ≤ 0.05 to declare the statistical significance of the variables.

### Ethics consideration

We obtained written consent of downloading and using SSA countries DHS dataset from the International Review Board of Demographic and Health Surveys (DHS) program data archivists after submitting a concept note and description of this study.

## Results

### Characteristics of the study participants

A total weighted sample of 77,001 children aged 6–23 months was included in this study. More than half (50.7%) of the children were male and 27,077(35.2%) were in the age group of 12–17 months. More than two-thirds of children (68.9%) were rural dwellers. The majorities of mothers were currently working 50,513 (65.6%) and married 53,880 (70.0%). Thirty-four thousand and two hundred twenty-nine (44.5%) of children were from households with poor wealth index. More than half (56.4%) of the children aged 6–23 months lived in a family size of ≥5. Around forty-nine thousand (63.9%) respondents have media exposure and distance of health facility was not a big problem to access medical care for 43,951(57.1%) respondents. Nearly half (47.9%) of children were 2^nd^ to 4^th^ birth order and most (79.3%) children were breastfeeding. The majority (83%) of children received vitamin-A in the first 2-months after delivery. The highest number 28,472 (37%) of children were from West Africa followed by east Africa 25,989 (33.75%) (Table 1).

**Table 1:** Socio-demographic characteristics of the children aged 6–23 months and respondents, demographic and health survey of sub-Saharan Africa (n = 77,001).

Variables	Category	Weighted Frequency	Prevalence
Child age in months	6–11	26,723	34.7%
	12–17	27,077	35.2%
	18–23	23,201	30.1%
Child sex	Male	39,011	50.7%
	Female	37,990	49.3%
Residence	Urban	23,989	31.2%
	Rural	53,013	68.9%
Mother’s age in years	< 20	11,845	15.4%
	20–34	50,692	65.8%
	35–49	14,465	18.8%
Mother’s educational level	No education	27,977	36.3%
	Primary	26,801	34.8%
	Secondary	19,452	25.3%
	Higher	2,772	3.6%
Mother’s occupation	Not working	26,488	34.4%
	Working	50,513	65.6%
Mother marital status	Married	53,880	70.0%
	Not married	23,121	30.0%
Father educational level	No education	32,342	42.0%
	Primary	20,582	26.7%
	Secondary	19,107	24.8%
	Higher	4,971	6.5%
Father occupation	Not working	13,260	17.2%
	Working	63,742	83.0%
Wealth index	Poor	34,229	44.5%
	Middle	15,602	20.3%
	Rich	27,170	35.3%
Family size	≤5	33,587	43.6%
	>5	43,414	56.4%
Media exposure	Yes	49,172	63.9%
	No	27,796	36.3%
Distance of health facility (getting medical help for self)	Big problem	33,051	42.9%
	Not a big problem	43,951	57.1%
Number of ANC	No at all	11,747	15.3%
	1–3	25,018	32.5%
	4–6	40,236	52.3%
Birth order	1	16,957	22.0%
	2–4	36,870	47.9%
	≥5	23,174	30.0%
Child is twin	Yes	2,322	3.0%
	No	74,678	97.0%
Current breast feeding	Yes	61,074	79.3%
	No	15,927	20.7%
Received vitamin-A in the first 2-monthes after delivery	Yes	13,079	17.0%
	No	63,922	83.0%
Child postnatal check within 2 months	Yes	26,988	35.0%
	No	50,013	65.0%
Had diarrhea recently	Yes	20,389	26.5%
	No	56,612	73.5%
Fever in the last two weeks	Yes	21,646	28.1%
	No	55,355	71.9%
Short rapid breath	Yes	9,339	12.3%
	No	67,662	87.9%
Drugs for intestinal parasites in last 6 months	Yes	26,209	34.0%
	No	50,792	66.0%
Sub-regions of sub-Saharan Africa countries	East Africa	25,989	33.8%
	West Africa	28,472	37.0%
	Central Africa	11,352	14.7%
	Southern Africa	11,188	14.5%

ANC: Antenatal care.

### Random effect analysis

In the null model, the values of Intra Class Correlation (ICC = 1.85%) and Median Odds Ratio (MOR = 1.08) implies the presence of clustering or community level variability of consumption of iron foods. Around 2% of the variation in consumption of iron-rich foods attributed to ICC. In the null model, the presence of heterogeneity of iron-rich food consumption between clusters was indicated by the MOR with a value of 1.08. It indicates that if we randomly select a 6–23 months old child, a child at the cluster with higher consumption of iron-rich foods had around 1.1 higher prevalence of consumption of iron-rich foods than a child at cluster with lower consumption of iron-rich food. Model III had the lowest deviance value (115,708.54) and hence it was selected as the best-fitted model (Table 2).

**Table 2:** Random effect analysis and model comparison results.

Parameters	Null model	Model I	Model II	Model III
**Community-level variance**	0.0062 (0.0038–0 .0104)	0.0009(0.00007–0.0122)	0.0041(0.002–0.009)	0.00019(1.56e-09–25.26)
**ICC**	1.85%	0.03%	0.12%	0.006%
**MOR**	1.082	1.029	1.062	1.013
**PCV**	Ref	85.484%	33.87%	96.94%
**Deviance(-2LL)**	121,795.72	117,424.38	120,125.23	115,708.54

ICC: Intra Class Correlation Coefficient, MOR: Median Odds Ratio, PCV: Proportional Change in Variance.

### Consumption of iron-rich foods and associated factors

The magnitude of iron-rich food consumption among children aged 6–23 months in SSA was 42.1% (95% CI: 41.78–42.48). Fish or shellfish was the most (15%) consumed food, while liver, heart, and other organs were the least (4.4%) consumed iron-rich foods (Table 3).

**Table 3:** Consumption of iron rich foods among children aged 6–23 months, sub-Saharan Africa (n = 77,001).

Variables	Category	Weighted Frequency	Percent(95%CI)
Iron rich food consumption in the last 24 hours	Good	32,440	42.1(41.78–42.48)
	Poor	44,561	57.8 (57.52–58.22)
Gave child egg in the last 24 hours	Yes	10,530	13.7
	No	66,471	86.3
Gave child meat (beef, pork, lamb, chicken, etc.) in the last 24 hours	Yes	12,257	15.9
	No	64,745	84.1
Gave child liver, heart, other organs in the last 24 hours	Yes	3,356	4.4
	No	73,645	95.6
Gave child fish or shellfish in the last 24 hours	Yes	20,117	26.1
	No	56,884	73.9

Both bivariable and multivariable multilevel Poisson regression analyses were employed to identify factors associated with iron-rich foods consumption. In the bivariable analysis, child age, birth order, current breastfeeding status, vitamin A intake, postnatal check within 2 months, fever, short rapid breath, use of drugs for intestinal parasites in last 6 months, maternal and paternal education and occupation, wealth index, family size, media exposure, the distance of health facility, number of ANC visit, residence and region of SSA were the candidate variables for the multilevel multivariable Poisson regression (p<0.25). Whereas, child age, current breastfeeding status, postnatal check within 2 months, use of drugs for intestinal parasites in last 6 months, maternal working status, maternal and paternal education, wealth index, media exposure, number of ANC visit, residence and region of SSA were significantly associated with good consumption of iron-rich foods in the multilevel multivariable Poisson regression (p≤ 0.05).

The prevalence of good consumption of iron among Children aged 12–17 and 18–23 months were 48% (adjusted prevalence ratio (aPR) = 1.48, 95% confidence interval (CI): 1.45–1.53), and 53% (aPR = 1.53, 95%CI: 1.49–1.58) higher, respectively, as compared with a child aged 6–11 months.

Children who were currently breastfeeding had a 14% (aPR = 1.14, 95%CI: 1.12–1.17) higher prevalence of good consumption of iron-rich foods compared to their counterparts. Child postnatal check within 2 months increased the likelihood of good consumption of iron-rich foods by 4% (aPR = 1.04, 95% CI: 1.01–1.06). Good consumption of iron-rich foods among 6–23 months aged children who took drugs for intestinal parasites in the last 6 months was 13% (aPR = 1.30, 95% CI: 1.11–1.15) higher as compared to those who didn’t take. Compared to children from women with no ANC visit, children from women with ANC visit of 1–3 and ≥4 had 17% (aPR = 1.17, 95% CI: 1.13–1.21), and 21% (aPR = 1.21, 95% CI: 1.17–1.25) higher consumption of iron-rich foods. Children from mothers with the educational level of primary, secondary and higher education had 14% (aPR = 1.14, 95% CI: 1.10–1.17), 18% (aPR = 1.18, 95% CI: 1.14–1.22), and 24%(aPR: 1.24, 95% CI: 1.17–1.30) higher prevalence of consumption of iron-rich foods, respectively, than children from non-educated mothers. The prevalence of good consumption of iron-rich foods among children from currently working mothers was 10% (aPR = 1.10, 95% CI: 1.08–1.13) higher than those of children from not currently working mothers. Primary, secondary, and higher educational level of the father was associated with 9% (aPR = 1.09, 95% CI: 1.06–1.12), 13% (aPR = 1.13, 95% CI: 1.10–1.17), and 15% (aPR = 1.15, 95% CI: 1.10–1.20) higher prevalence of children’s good consumption of iron-rich foods, respectively, than that of children from non-educated fathers. Children from a family with rich and middle wealth index had 6% (aPR = 1.06, 95%CI: 1.03–1.09), and 14% (aPR = 1.14 95% CI: 1.10–1.18) higher prevalence of good consumption of iron-rich foods than children from a family with poor wealth index.

Children from the urban area had 14% (aPR = 1.14, 95% CI: 1.11–1.18) higher prevalence of consuming iron-rich foods than a child from a rural area. Children from a woman with Media exposure had 15% (aPR = 1.26, 95%CI: 1.12–1.17) higher prevalence of consumption of iron-rich foods as compared to children from a woman with no media exposure. Children from West Africa, Central Africa, and Southern Africa countries had 31% (aPR = 1.31, 95% CI: 1.26–1.35), 48% (aPR = 1.48, 95% CI: 1.42–1.53), and 52% (aPR = 1.52, 95% CI: 1.46–1.57) higher prevalence of good consumption of iron-rich foods, respectively, than children from east Africa countries (Table 4).

**Table 4:** Multilevel Poisson regression analyses of good consumption of iron in sub-Saharan Africa (n = 77,001).

Variables		Good consumption		Prevalence Ratio (95% Confidence Interval)	
		Yes	No	uPR	aPR
		N (%)	N (%)		
Child age in months	6–11	8,090(24.94)	18,633(41.81)	1	1
	12–17	12,624(38.91)	14,453(32.43)	1.53(1.49–1.56)	1.48(1.45–1.53)*
	18–23	11,726(36.15)	11,476(25.75)	1.66(1.63–1.70)	1.53(1.49–1.58)*
Child sex	Male	16,482 (50.81)	15,958(49.19)	1.00(0.98–1.02)	
	Female	22,529 (50.56)	22,032(49.44)	1	
Residence	Urban	13,319(41.06)	10,670 (23.94)	1.44(1.41–1.48)	1.14(1.11–1.18)*
	Rural	19,121(58.94)	33,892(76.06)	1	1
Mother’s age in years	< 20	4,668(14.39)	7,176(16.10)	1	1
	20–34	21,744(67.03)	28,948(64.96)	1.07(1.05–1.09)	1.05(0.99–1.05)
	35–49	6,028(18.58)	8,437(18.93)	1.05(1.0–1.07)	1.03(0.98–1.087)
Mother’s educational level	No education	9,138(28.17)	18,839(42.28)	1	1
	Primary	10,956 (33.77)	15,845(35.56)	1.25(1.22–1.28)	1.14(1.10–1.17)*
	Secondary	10,567(32.58)	8,884(19.94)	1.65(1.60–1.69)	1.18(1.14–1.22)*
	Higher	1,779(5.48)	993(2.23)	1.90(1.83–1.98)	1.24(1.17–1.30)*
Mother’s Occupation	Not working	10,662(32.87)	15,826(35.51)	1	1
	Working	21,778(67.13)	28,736(64.49)	1.07(1.05–1.09)	1.10(1.08–1.13)*
Father Educational level	No education	11,753(36.23)	20,589(46.20)	1	1
	Primary	7,878 (24.28)	12,704(28.51)	1.07(1.05–1.10)	1.09(1.06–1.12)*
	Secondary	9,883(30.46)	9,224(20.70)	1.42(1.39–1.45)	1.13(1.10–1.17)*
	Higher	2,927(9.02)	2,045(4.59)	1.57(1.52–1.62)	1.15(1.10–1.20)*
Father Occupation	Not working	5,968 (18.40)	7,292(16.36)	1	1
	Working	26,472(81.60)	37,270(83.64)	0.93(0.91–0.94)	0.94(0.92–0 .97)
Wealth Index	Poor	11,866(36.58)	22,363 (50.18)	1	1
	Middle	6,420(19.79)	9,182(20.61)	1.21(1.18–1.24)	1.06(1.03–1.09)*
	Rich	14,154(43.63)	13,016(29.2)	1.47(1.43–1.50)	1.14(1.10–1.18)*
Family Size	≤5	14,498(44.69)	19,088 (42.84)	1.03(1.02–1.05)	0.98(0.96–1.00)
	>5	17,941(55.31)	25,473(57.16)	1	1
Media Exposure	Yes	23,244(71.68)	25,928(58.21)	1.41(1.38–1.43)	1.15(1.12–1.17)*
	No	9,186(28.32)	18,611(41.79)	1	1
Distance of health facility (getting medical help for self)	Big problem	12,784(39.41)	20,266(45.48)	1	1
	Not a big problem	19,656(60.59)	24,295(54.52)	1.17(1.15–1.19)	1.01(0.99–1.04)
Birth order	1	7,615 (23.47)	9,342(20.97)	1	1
	2–4	16,035(49.43)	20,835(46.76)	0.96(0.96–0 .99)	0.95(0.95–1.00)
	≥5	8,790(27.10)	14,384(32.28)	0.87(0.85–0.89)	0.97(0.93–1.00)
Child is Twin	Yes	997(3.07)	1,326(2.97)	0.99(0.93–1.05)	
	No	31,442(96.93)	43,236(97.03)	1	
Number of ANC	No at all	3,830(11.81)	7,917(17.77)	1	1
	1–3	9,542(29.41)	15,476(34.73)	1.18(1.15–1.22)	1.17(1.13–1.21)*
	≥4	19,068(58.78)	21,168(47.50)	1.45(1.40–1.49)	1.21(1.17–1.25)*
Current Breast Feeding	Yes	23,074(71.13)	37,999(85.27	1	1
	No	9,365(28.87)	6,562(14.73)	1.54(1.51–1.57)	1.14(1.12–1.17)*
Received vitamin-A in the first 2-monthes after delivery	Yes	6,056(18.67)	7,023(15.76)	1.11(1.09–1.14)	1.00(0.98–1.03)
	No	26,384(81.33)	37,538(84.24)	1	1
Child postnatal check within 2 months	Yes	12,641(38.97)	14,347(32.20)	1.17(1.15–1.19)	1.04(1.01–1.06)*
	No	19,799(61.03)	30,215(67.80)	1	
Had diarrhea recently	Yes	8,592(26.48)	11,798(26.47)	1	
	No	23,848(73.52)	32,764(73.53)	1.00(0.99–1.03)	
Fever in the last two weeks	Yes	8,936(27.55)	12,710(28.52)	1	1
	No	23,503(72.45)	31,852(71.48)	1.03(1.01–1.04)	0.99(0.97–1.02)
Short rapid breath	Yes	3,790(11.68)	5,549(12.45)	1	1
	No	28,650(88.32)	39,012(87.55)	1.04(1.01–1.07)	1.00(0.97–1.04)
Drugs for intestinal parasites in last 6 months	Yes	13,301(41.00)	12,909(28.97)	1.36(1.33–1.39)	1.13(1.11–1.15)*
	No	19,139(59.00)	31,653(71.03)	1	1
Sub-Region of sub-Saharan Africa	East Africa	8,628(26.60)	17,361 (38.96)	1	1
	West Africa	11,931(36.78)	16,541(37.12)	1.22(1.19–1.25)	1.31(1.26–1.35)*
	Central Africa	5,768(17.78)	5,583(12.53)	1.49(1.44–1.53)	1.48(1.42–1.53)*
	Southern Africa	6,113(18.84)	5,075(11.39)	1.56(1.51–1.62)	1.52(1.46–1.57)*

*p ≤ 0.05, uPR: Unadjusted Prevalence Ratio, aPR: Adjusted Prevalence Ratio, CI: Confidence Interval.

## Discussion

Iron-rich foods consumption has a significant role in blood iron level and anemia status. However, there is no evidence of the consumption of iron-rich food status and its associated factors in SSA. Hence, this study determined the weighted prevalence of good consumption of iron-rich foods and its associated factors among children aged 6–23 months in SSA.

The weighted prevalence of good consumption of iron-rich foods among children aged 6–23 months in SSA was 42.1%, which is lower than the result of studies conducted in Australia (82.6%) [28], Ireland (90%) [29], Mexico (63.1%) [30], East Asia and the Pacific (62.5%) [31], China (51%) [21], and Bangladesh (50%) [32]. The lower prevalence of good consumption of iron-rich foods in SSA might be due to household’s food insecurity and poor economic status of SSA countries, the poorest in the world, which makes iron-rich foods or animal source foods unaffordable [33] while such foods are greatly accessible in high-income counties [23, 34]. Children 6–23 months in most regions of SSA consumed no animal source food, iron-rich foods [31], instead tubers and cereals are the most common food [35] and consumption of meat in Africa is the lowest in the world, influenced by economic, cultural, and religious factors [36].

In light of this low prevalence of consumption of iron-rich foods among children aged 6–23 months in SSA, it is imperative for nutrition programs to emphasize and improve consumption of iron rich foods, animal source foods, and other alternative foods rich in iron (vegetables and fruits) in SSA young children.

However, the prevalence of good consumption of iron-rich foods in the present study is higher than the prevalence reported in Zambia (33.1%) [37], Ethiopia (21.4%) [24], and Madagascar (19.6%) [38]. This divergent result could be due to a difference in socioeconomic status, beliefs, norms, and cultural practice of children feeding. In Ethiopia, animal source foods are consumed mostly only during holidays as they are considered luxurious foods instead of basic daily requirements. Moreover, animal and animal source foods rich in iron are used mainly for the market purpose [39]. In the case of Zambia, most (70%) of energy source is maize with low consumption of iron rich foods [33].

Individual and community-level variables were found to be associated with the consumption of iron rich foods. Children aged 12–23 months had higher prevalence of good consumption of iron-rich foods than children aged from 6–11 months. This is in line with studies elsewhere [31, 35, 40–42]. This lower consumption of iron-rich food in 6–12 months children as compared to older children might be due to the mother’s perceptions that children before the age of 1 year should not consume animal-source foods [43]. Moreover, older children have regular consumption of foods prepared for the family with a greater content of iron [44]. Therefore, there is a need for national strategies to enhance the consumption of iron-rich foods in infants.

As this study demonstrated, the use of drugs for intestinal parasites in the last 6 months for children aged 6–23 months was associated with higher prevalence of good consumption of iron-rich foods than those who didn’t use. This finding is in agreement with the study in Uganda [45]. This might be because women who use drugs for intestinal parasites for their children will have similar motivation and commitment to give iron-rich foods for their children and an opportunity to interact with health care providers, and hence get counseling on appropriate child nutrition. Moreover, taking drugs for intestinal parasites might increase children’s food intake, thereby increases the consumption of iron-rich foods [46].

Children from mothers and fathers with educational status of primary or higher had higher prevalence of good consumption of iron-rich foods than children whose parents were non-educated. This finding is in agreement with other studies [41, 47–51]. The higher odds of good consumption of iron-rich foods in children from educated parents might be due to better exposure to media, understanding of and more access to information about children’s feeding practices and consumption of iron-rich foods [51]. Moreover, higher education is also related to higher income that in turn allows them to afford more iron-rich foods, like meat.

In the present study children with a postnatal check (PNC) within 2 months had higher prevalence of good consumption of iron-rich foods which is in line with another study [35]. In agreement with the previous studies [41, 42, 52], in this study, children from mothers with ANC visits had higher prevalence of good consumption of iron-rich foods than their counterparts. The possible justification for the higher odds of good consumption of iron-rich foods among children with a postnatal check within 2 months and from the mothers with ANC visit might be because, during the ANC and PNC visits, mothers will have an opportunity to learn about healthy child nutrition and appropriate feeding practice and get motivated to nourish their children iron-rich foods [35]. Therefore it is imperative to promote community-based nutritional education, and offer universal ANC and PNC for mothers in SSA countries to improve consumption of iron-rich foods consumption.

Wealth index was the other factor that significantly associated with good consumption of iron-rich foods. In line with this study, studies elsewhere revealed the association of high wealth index with good consumption of iron-rich foods [31, 35, 41, 53–55]. This is obviously due to the high economic status resulting in better access to financial resources and information to afford iron-rich foods. It is revealed that a lack of financial resources is a barrier to access nutritious foods [35, 56]. Therefore, especially for low income households, it is imperative to give attention to other less expensive and easily affordable iron rich foods like vegetables and fruits.

Children from mothers with media exposure had higher prevalence of good consumption of iron-rich foods than the children whose mothers haven’t media exposure. It is in agreement with other studies [38, 57–59]. The association between mass media exposure and good consumption of iron-rich foods might be because of exposure to mass media, a great source of information for the society, has been found highly important in improving the knowledge and practices of mothers on infant and young child feeding by increasing caregivers access to health message on appropriate feeding practice of their children [60, 61].

The strength of this study, the first study to assess consumption of iron-rich foods among children aged 6–23 months at SSA level, includes; it uses large representative sample for children aged 6–23 months from the latest DHS data of SSA countries, and hence it is generalizable. This study assessed the association between individual, and community level variables and consumption of iron-rich foods using a multilevel Poisson regression model that accounts for the correlated nature of DHS data to get reliable estimates. However, the following limitations should be considered. First, this study couldn’t show casual associations because the data source is a cross-sectional survey. Second, there might be a recall bias as data was collected from the mother or the caregiver by interview. Third, since we have used secondary data, DHS data, consumption of plant-based iron rich foods and iron-fortified foods were not considered, which might have underestimated the prevalence of consumption of iron rich foods in our study. Moreover, their bioavailability was not assessed. Though the iron rich foods are consumed, their bioavailability is affected by concomitant dietary compositions that are either Fe absorption enhancers (ascorbic acid) or inhibitors (tea polyphnols). However, DHS didn’t consider them. Lastly, quantities of iron-rich foods consumed or adequacy of iron intake, according to WHO recommended daily allowance of iron, was not determined.

## Conclusion

The prevalence of good consumption of iron-rich foods among children aged 6–23 months in SSA countries is low. Child factors: age, current breastfeeding status, drugs use for intestinal parasites in last 6 months; family factors; maternal and paternal education, maternal occupation, postnatal check within 2 months wealth index, media exposure, number of ANC visit; and community level factors: residence and sub-region of SSA were significantly associated with consumption of iron-rich foods. Strategies to increase consumption of iron-rich foods during this critical stage, the first two years of life, of growth and development should be designed by considering these modifiable factors for children in SSA. Moreover, strategies that allow the improvements of consumption of iron rich foods from other sources, instead of thinking only animal source foods, should be designed. We strongly recommend the forthcoming researchers to consider and investigate plant-based iron rich foods and bioavailability of iron rich foods using a primary data.

## List of abbreviations

ANC: Antenatal Care
ID: Iron deficiency
IDA: Iron Deficiency Anemia
PNC: Postnatal Check
SSA: sub-Saharan Africa
WHO: World Health Organization
